# Supplementary material for: Risk of SARS-CoV-2 Reinfections in a Prospective Inception Cohort Study: Impact of COVID-19 Vaccination
Source: J Clin Med. 2022 Jun 10;11(12):3352. doi: 10.3390/jcm11123352 (PMC9225121; doi:10.3390/jcm11123352)
Supplement: Supplementary file 1 [file jcm-11-03352-s001.zip › jcm-1732020-supplementary.pdf]

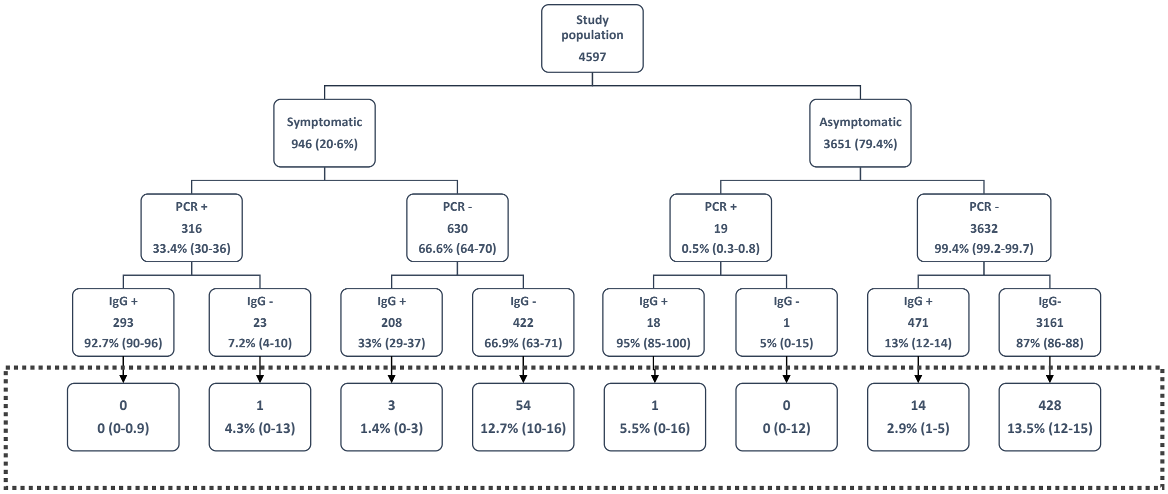

Figure S1. Flow diagram of the 4597 HCWs with follow-up until individual vaccination date, classified according to the presence of symptoms, PCR result, and specific serology result (IgG+/IgG-), and the rate of new infections/reinfections (black box). Between parentheses, 95% confidence interval for each rate.

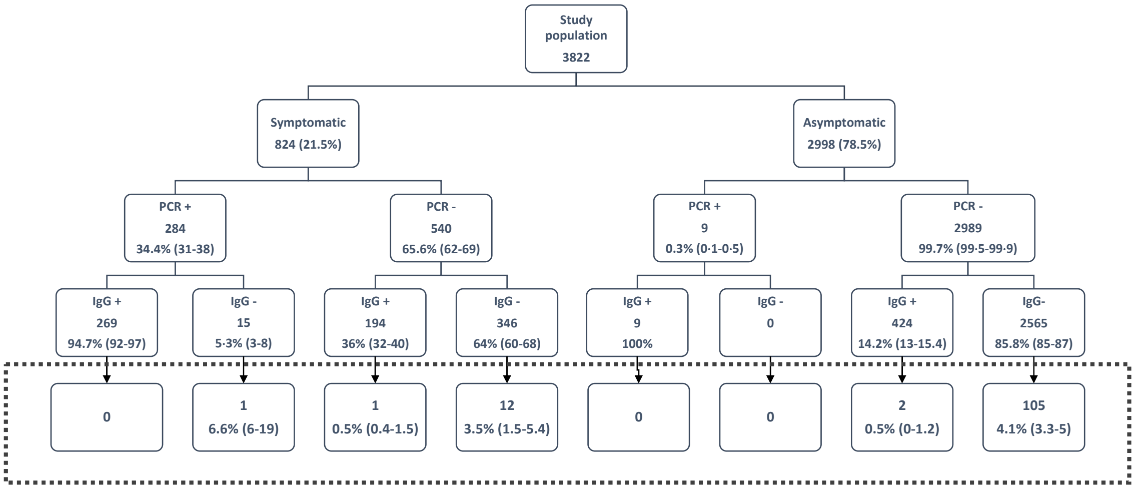

Figure S2. Flow diagram of the HCWs followed since individual vaccination until 15 November 2021, according to previous presence of symptoms, PCR result and specific serology (IgG+/IgG-), and rate of new infections/reinfections (black box). Between parentheses, 95% confidence interval for each rate.
